# ASCO 2016 – update colorectal liver metastases

**DOI:** 10.1007/s12254-016-0308-y

**Published:** 2017-01-17

**Authors:** Klaus Kaczirek

**Affiliations:** Department of Surgery and Center for Perioperative Medicine, Medical University of Vienna, Waehringer Guertel 18–20, 1090 Vienna, Austria

**Keywords:** Colorectal liver metastasis, Surgery, Hepatectomy, ASCO, Chemotherapy

## Abstract

This short review summarizes the most relevant data on colorectal liver metastases (CLM) presented at the 2016 Annual Meeting of the American Society of Clinical Oncology (ASCO). For this update, congress abstracts were grouped into three clinically relevant scenarios: CLM that are deemed upfront resectable, borderline resectable, and primarily unresectable. According to a large registry study, small, upfront resectable, solitary CLM should be resected using a parenchymal sparing technique. A prospective noninterventional study suggested that chemotherapy plus the anti-VEGF (vascular endothelial growth factor) antibody bevacizumab is effective and well tolerated in patients with borderline resectable CLM. In the setting of CLM deemed initially unresectable, a phase 2 study reported superior outcomes in terms of liver metastases resection rates as well as overall survival (OS) in patients receiving a chemotherapy triplet (FOLFIRINOX) plus a targeted agent as compared to FOLFIRI or FOLFOX4 with the same targeted therapy. Finally, a study suggested that tailoring first-line chemotherapy according to *TYMS-3’UTR* and *ERCC1-118* polymorphism status results in higher response rates and longer progression-free survival (PFS) as well as OS.

## Review

In patients presenting with colorectal cancer liver metastases (CLM), three clinical scenarios can be distinguished: CLM that are deemed upfront resectable, borderline resectable lesions, and initially unresectable metastases that could be amendable to curative resection in patients responding to induction chemotherapy. The term “borderline resectable” is commonly defined as metastases potentially amendable to curative resection but surgery of these lesions is regarded technically and/or biologically more challenging than in upfront resectable CLM.

At the 2016 ASCO Annual Meeting, one abstract was presented on the treatment of *upfront resectable CLM* [1]. This study evaluated surgical as well as long-term outcomes in patients with solitary small metastases in the right liver who underwent either parenchymal-preserving hepatectomy (PH) or anatomical right hepatectomy (RH). Data were extracted from a large registry (LiverMetSurvey) including 21,072 resections performed between 2000 and 2015. A total number of 1720 patients met the inclusion criteria for this analysis; of these, 1478 (86%) were treated with PH, while 242 (14%) underwent RH. Patients in the RH group had larger metastases, a higher percentage of initially unresectable disease, a lower rate of laparoscopic resections and fewer patients received postoperative chemotherapy as compared to the PH group. Not surprisingly, PH was superior in terms of number of blood transfusions required, duration of in-hospital stay, rate of major complications as well as 90-day mortality rate while repeat hepatectomy was less frequently performed after RH. Despite adverse clinical features as outlined, there was no difference in recurrence-free survival, time to last unresectable recurrence, and OS between the two groups. When patients with liver-only recurrences, however, were analyzed separately, time to last unresectable recurrence and OS was significantly longer in the PH group. In a multivariate model, nodal status (RR 0.47; 95% CI 0.23–0.95), CEA levels <15 ng/ml (RR 1.85; 95% CI 1.01–3.37), and PH (RR 3.7; 95% CI 1.77–7.72) were significantly associated with superior 5‑year OS. Although these data were extracted from a registry and this is not a randomized study, this study suggests that PH should be regarded as a standard-of-care for solitary small CLM.

Patients with *borderline resectable metastases* were included into a multicenter, prospective noninterventional study [2]. Patients received first line chemotherapy (mostly FOLFOX or FOLFIRI) in combination with bevacizumab, a humanized monoclonal antibody targeting VEGF. A total number of 218 patients were included with 205 evaluable for outcome analysis. The primary objective was the rate of patients without detectable metastatic disease (no evidence of disease; NED) with or without secondary resection. Overall, 86% of patients had liver-only disease, and 104 patients (51%) were resected after first-line chemotherapy. NED was achieved in 92 patients (48%; 88 with surgery and 4 with chemotherapy alone). Progression-free survival (PFS) was 15.7 months in patients with NED as compared to 11.5 months in patients without; OS at 36 months was 77% and 52%, respectively. Therefore, this real-life study suggests that chemotherapy plus bevacizumab is an effective and well-tolerated treatment option in patients with borderline resectable CLM.

In the setting of patients with CLM deemed *initially as unresectable,* the most relevant study was a phase 2 trial evaluating different chemotherapy backbones for induction therapy [3]. In all, 256 patients were randomized to receive a chemotherapy doublet (either FOLFIRI or FOLFOX4, *n* = 126) or triplet (FOLFIRINOX, *n* = 136) in combination with either bevacizumab or cetuximab according to their respective *kRAS* mutations status. The main objective of the trial was the resection rate in both groups; of note, due to the phase II design and stratification to either bevacizumab or cetuximab patient subgroups were rather small.

Triplet chemotherapy increased resection rates from 45.2 to 56.9%(*p* = 0.062) and the combination of chemotherapy with cetuximab yielded numerically higher resection rates as compared to bevacizumab (55.6% versus 44.7%; *p* = 0.087). Severe adverse events were not significantly different between groups. Importantly, median OS was significantly longer in patients receiving FOLFIRINOX (not reached versus 36 months, respectively; *p* = 0.048). The authors therefore concluded that the combination of FOLFIRINOX with targeted therapy was associated with better OS and liver metastases resection rates than either FOLFIRI or FOLFOX4 with targeted therapy.

High response and conversion rates (secondary endpoints) were also described in a study that investigated modified FOLFOXIRI plus cetuximab followed by cetuximab or bevacizumab maintenance [4] although neither arm met the predefined primary endpoint.

Another abstract dealt with the pharmacogenetic selection of chemotherapy in unresectable CLM [5]. The design of this phase 2 study was based on earlier results suggesting that patients harboring *TYMS*-3’UTR +6 bp/+6 bp and *ERCC1*–118 C/T or C/C genotypes may derive greater benefit from capecitabine as compared to 5‑fluorouracil (FU) as part of an oxaliplatin-based first-line regimen. Overall, 180 patients were randomized to a control arm (60 patients receiving XELOX plus bevacizumab) and an experimental arm (120 patients receiving bevacizumab plus FUOX, XELOX, FUIRI, or XELIRI based upon their pharmacogenetic profile). The combined rates of complete and partial response were 48% in the control group and 65% in the experimental group (*p* = 0.04). In total, 37 patients were converted to resectable status of liver metastases, the R0 surgery rate was 43% (7/16) in the control arm and 86% (18/21) in the experimental arm (*p* = 0.018); PFS and OS were superior in the experimental arm as well. The authors concluded that prospective selection of first-line treatment according to *TYMS*-3’UTR and *ERCC1-118* polymorphisms should be taken into account in order to increase the probability of R0 surgery.

### Take home message

Parenchymal sparing resections should be performed for solitary, small colorectal liver metastases. In case of unresectable metastases high conversion rates can be achieved by using chemotherapy triplets such as FOLFIRINOX or FOLFOXIRI with either cetuximab or bevacizumab. Pharmacogenetic selection of chemotherapy may additionally improve outcomes in this group of patients.

## References

[1] Hosokawa I, Allard MA, Nitta H (2016). Oncological benefit of parenchyma-preserving hepatectomy for colorectal liver metastasis. J Clin Oncol.

[2] Malka D, Metges J, Elias D (2016). Bevacizumab plus chemotherapy (bev/CT) as first-line therapy for patients with potentially resectable metastatic colorectal cancer (mCRC): Final results of the French noninterventional PICASSO study. J Clin Oncol.

[3] Ychou M, Rivoire M, Thezenas S (2016). FOLFIRINOX combined to targeted therapy according RAS status for colorectal cancer patients with liver metastases initially non-resectable: a phase II randomized study – Prodige 14 – ACCORD 21 (METHEP-2), a unicancer GI trial. J Clin Oncol.

[4] Antoniotti C, Cremolini C, Loupakis F (2016). Modified FOLFOXIRI (mFOLFOXIRI) plus cetuximab (cet), followed by cet or bevacizumab (bev) maintenance, in RAS/BRAF wild-type (wt) metastatic colorectal cancer (mCRC): results of the phase II randomized MACBETH trial by GONO. J Clin Oncol.

[5] Abad A, Vieitez J, Alonso V (2016). Effect of pharmacogenetic-based selection of first-line chemotherapy on response rate and R0 surgery in metastatic CRC patients. J Clin Oncol.

